# Disease Modifying Therapies for Alzheimer's Disease Targeting A****β**** Oligomers: Implications for Therapeutic Mechanisms

**DOI:** 10.1155/2013/984041

**Published:** 2013-08-26

**Authors:** Etsuro Matsubara, Ayumi Takamura, Yasuhide Okamoto, Hideto Oono, Takashi Nakata, Yasuhito Wakasaya, Takeshi Kawarabayashi, Mikio Shoji

**Affiliations:** ^1^Department of Neurology, Institute of Brain Science, Hirosaki University Graduate School of Medicine, Aomori 036-8562, Japan; ^2^Shin-Yurigaoka General Hospital, Kanagawa 215-0026, Japan; ^3^Immunas Pharma Incorporation, Kanagawa 213-0012, Japan

## Abstract

Several lines of evidence indicate that amyloid **β** (A**β**), particularly A**β** oligomers (A**β**Os), plays a causative role in Alzheimer's disease. However, the mechanisms underlying the action of an anti-A**β**O antibody to clarify the toxic action of A**β**Os remain elusive. Here, we showed that the anti-A**β**O antibody (monoclonal 72D9) can modify the A**β** aggregation pathway. We also found that 72D9 directly sequesters both extracellular and intraneuronal A**β**Os in a nontoxic state. Thus, therapeutic intervention targeting A**β**Os is a promising strategy for neuronal protection in Alzheimer's disease.

## 1. Introduction

Disease modifying therapies for Alzheimer's disease (AD) are based on strategic approaches towards the “amyloid cascade hypothesis” [1]. Among them, what appears to meet our expectation is A*β* immunotherapy, which involves immune-mediated A*β* depletion in the brain [2–5]. However, recent phase III trials of certain A*β* monoclonal antibodies (e.g., bapineuzumab and solanezumab) failed to significantly slow cognitive and functional declines. Despite the disappointing outcomes, the effects of both antibodies were confirmed on biomarkers (http://www.alzforum.org/new/detail.asp?id=3268, http://www.alzforum.org/new/detail.asp?id=3442). From this point of view, A*β* immunotherapy is now considered as prophylaxis for patients with mild cognitive impairment. Indeed, solanezumab is now being evaluated in terms of its efficacy for antiamyloid treatment in an asymptomatic Alzheimer's disease (A4) prevention clinical trial (http://www.alzforum.org/new/detail.asp?id=3379).

Accumulated lines of evidence indicate that memory loss represents a synaptic failure caused directly by soluble A*β*Os [6–9] and that amyloid fibrils may cause neuronal injury indirectly via microglial activation [10]. Thus, the classical amyloid cascade hypothesis [1] underwent a modification in which emphasis was switched to intermediate forms of A*β* such as A*β*Os [11–15], rather than fibrillar A*β* [10]. Therapeutic intervention targeting A*β*Os alone should be a promising strategy for AD treatment [16, 17]. Several major hypotheses underlying the action of A*β* immunotherapy have been proposed, including phagocytosis by microglia [18], peripheral sink [19], neonatal Fc receptor (FcRn) mediated A*β* transport across the blood-brain barrier (BBB) [20], catalytic modification of A*β* fibrils [21], intracerebral sequestration of A*β* in a monomeric state [22], and antibody-mediated neutralization of A*β*Os and/or tau toxicity [23]. However, the precise molecular mechanisms underlying disease modifying therapy targeting A*β*Os remain elusive. Here, among the abovementioned hypothetic mechanisms, the last issue with particular emphasis on the action of an anti-A*β*O antibody was evaluated. We found that the anti-A*β*O antibody (72D9) can modify the A*β* aggregation pathway and that it directly sequesters both extracellular and intraneuronal A*β*Os in a nontoxic state. 

## 2. Materials and Methods

### 2.1. Antibodies

Monoclonal 72D9 was generated and characterized as described previously [17]. Polyclonal A11 specific to A*β*Os was purchased from BioSource (Camarillo, CA, USA). Goat anti-mouse IgG conjugated with Alexa Fluor (AF) 488 or 594 and goat anti-rat IgG conjugated with AF 488 were purchased from Molecular Probes (Eugene, OR, USA). Anti-mouse IgG2b (the IgG2b isotype) was purchased from Sigma (St. Louis, MO, USA). 

### 2.2. A*β* Incubation and ThT Assay

ThT assay was performed as described previously [24]. A*β* solutions at 12.5 *μ*M were incubated with Abs (72D9 and IgG2b) at the indicated concentration and at 37°C for 24 h. The ThT fluorescence intensity in the incubation mixtures was determined using a spectrofluorophotometer (RF-5300PC) (Shimadzu Co., Kyoto, Japan). The optimum fluorescence intensity of amyloid fibrils was measured at excitation and emission wavelengths of 446 and 490 nm, respectively, with a reaction mixture (1.0 mL) containing 5 *μ*M ThT and 50 mM glycine-NaOH at pH 8.5. Fluorescence intensity was measured immediately after preparing the mixture.

### 2.3. A*β*-Induced Toxicity Assay

We conducted the A*β*-induced toxicity assay in the presence or absence of Abs according to previously published methods [24]. Briefly, human neuroblastoma SH-SY5Y cells were cultured in DMEM (Invitrogen, Carlsbad, CA, USA) supplemented with 10% heat-inactivated horse serum (Invitrogen) and 5% FBS (Invitrogen). Basically, toxicity was assessed using A*β*1-42 at 12.5 *μ*M with (0, 0.5, 1.0, and 1.5 *μ*M) and without Abs for 24 h at 37°C. Toxicity was assessed by LDH assay in accordance with the manufacturer's instructions (Molecular Probes, Eugene, OR, USA). 

### 2.4. Electron Microscopy (EM)

For electron microscopy, samples were diluted with 0.1% distilled ammonia solution and spread on carbon coated grids. The grids were negatively stained with 1% phosphotungstic acid and examined under a Hitachi H-7000 electron microscope (Tokyo, Japan) at an acceleration voltage of 77 kV. 

### 2.5. Double Immunolabeling and Confocal Laser Microscopy

To elucidate the ability of 72D9 for the intracerebral sequestration of A*β*Os, paraffin-embedded mouse brain sections from 72D9- and IgG2b-treated 3xTg-AD mice (*n* = 6, each) [17] were immunolabeled with Alexa Fluor-conjugated secondary antibodies (green). A*β*Os were immunolabeled with A11, which was detected with Alexa Fluor-conjugated secondary antibodies (red), and nucleases, with the antibody against DAPI, which was detected with Alexa Fluor-conjugated secondary antibodies (blue). Sections were imaged using a confocal laser scanning microscope (Carl Zeiss LSM510). 

## 3. Results and Discussion

### 3.1. Modification of the A*β* Aggregation Pathway

**Figure 1:**
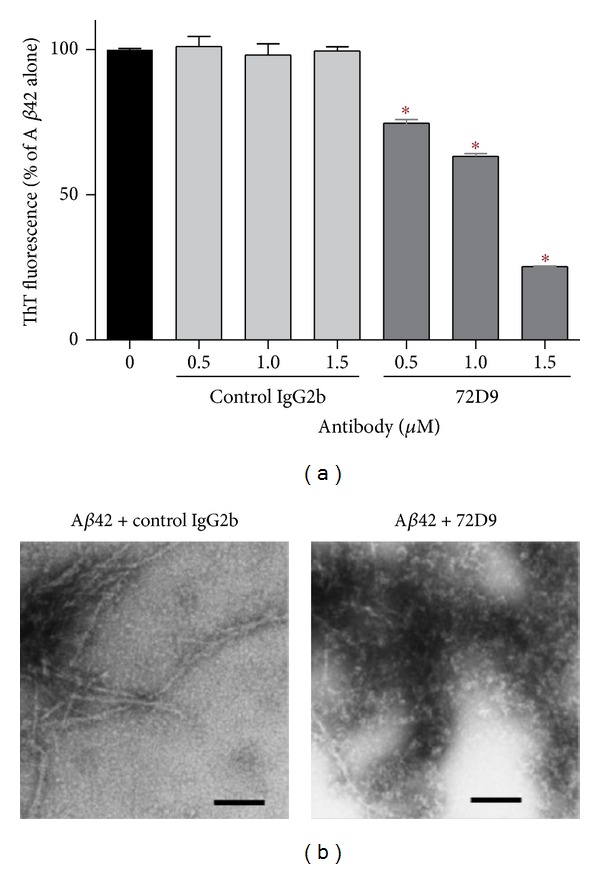
Antifibrillogenic activities of 72D9. (a) Fibril formation of A*β*1-42 at 12.5 *μ*M was assayed on the basis of ThT fluorescence intensity at 37°C for 24 h: dose-dependent inhibition of A*β*1-42 assembly was observed for 72D9; however, nonspecific IgG2b failed to inhibit seed-free A*β*1-42 (540,000 ×g ThT-negative supernatants) assembly. (b) Electron micrographs of incubation mixture containing 50 *μ*M A*β*1-42 preincubated with IgG2b or 72D9. A*β*1-42 with control IgG2b shows mature fibrils (left panel). A*β*1-42 with 72D9 shows nonfibrillar amorphous structures (right panel). Scale bar = 200 nm. Experimental results were analyzed with one-way ANOVA, followed by the Tukey test for post hoc analysis: statistical significance compared with A*β*1-42 alone (*P < 0.0001).

Our previous *in vivo* experiments using 72D9 resulted in a marked reduction in the density of Gallyas-Braak positive senile plaques in 3xTg-AD mice with improved cognition [17]. Since 72D9 does not recognize A*β* fibrils, microglial phagocytosis was not observed [17], indicating that 72D9 can modify the A*β* aggregation pathway *in vivo*. To assess this issue, we incubated 12.5 *μ*M seed-free A*β*42 alone or with antibodies at 37°C for 24 h. As shown in Figure 1(a), ThT fluorescence intensity decreased with the increasing 72D9 concentration, and nonspecific IgG2b showed no antifibrillogenic activity. Using EM (Figure 1(b)), we find A*β* fibrils in the presence of IgG2b; however, a mixture of A*β* fibrils and nonfibrillar amorphous A*β* structures was observed in the presence of 72D9. In support of our findings, a similar modification of the A*β* aggregation pathway using antibody fragments is reported by three groups, who proposed that antibody fragments withdraw A*β*Os from the A*β* amyloid fibril-forming pathway, maintaining them in nonfibrillar amorphous structures [25–28]. From a structural viewpoint, it has been shown that bapineuzumab captures A*β* in a monomeric helical conformation at the N-terminus [29]. Another intracerebral sequestration of A*β* in a monomeric state to prevent further A*β* assembly and related neurotoxicity is also reported by m266.2, a parent of the humanized monoclonal antibody solanezumab [22]. However, these two mechanisms are not the case for 72D9, because 72D9 does not recognize A*β* monomers [17]. Thus, our data indicate that 72D9 prefers to lead A*β*Os to form nonfibrillar amorphous structures in a chaperone-like manner, which allow A*β*Os to exist in a nontoxic state. 

### 3.2. Intracerebral Sequestration of A*β*Os in a Nontoxic State

**Figure 2:**
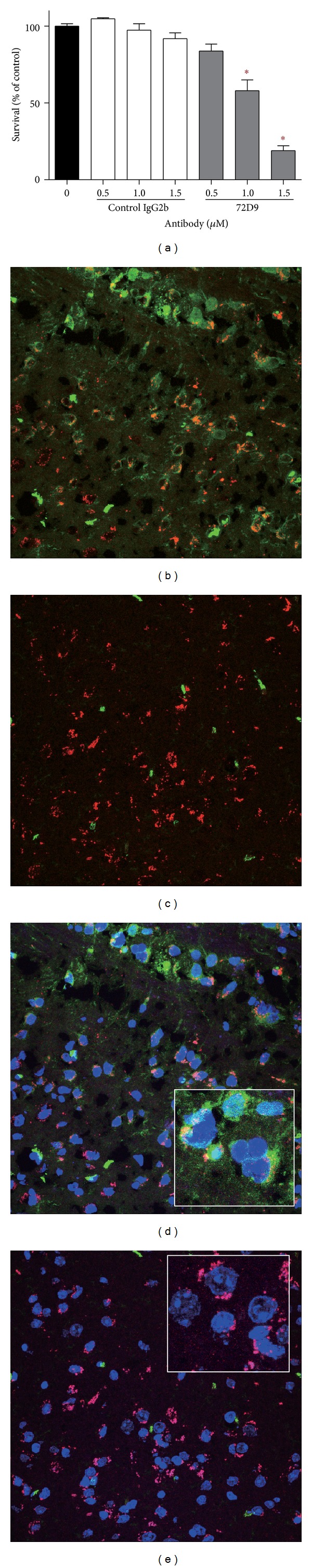
Antitoxic activity of  72D9. (a) SH-5YSY cells were exposed to 12.5 *μ*M seed-free A*β*42 with control IgG2b or 72D9 at 37°C for 24 h. Level of LDH released from SH-SY5Y cells treated for 24 h with 12.5 *μ*M A*β*1-42 with control IgG2b or 72D9 at the indicated concentrations (0, 0.5, 1.0, and 1.5 *μ*M). Each value indicates the percent level of LDH released following treatment with incubation mixtures relative to the level of LDH released following treatment with Triton X-100. Each column indicates average ± SD. The *P* value was determined by one-way ANOVA, followed by Tukey test for post hoc analysis: statistical significance compared with A*β*1-42 alone (*P < 0.0001). (b) Sections of control 72D9-treated or IgG2b-treated 3xTg-AD mouse brain were analyzed by immunofluorescence imaging of 72D9 (green), polyclonal A11 (red), and DAPI (blue). Inset: representative higher magnification images are shown in the insets of panels (d) and (e).

From the abovementioned functional viewpoint on A*β*Os, we further characterized the antitoxic activity of 72D9 *in vitro*. SH-5YSY cells were incubated at 37°C for 24 h with 12.5 *μ*M seed-free A*β*42 with or without antibodies. Compared with vehicle treatment, LDH assay of SH-SY5Y cells revealed significant neuronal death in the presence of nonspecific IgG2b (Figure 2(a)). In contrast, monoclonal 72D9 afforded nearly complete blockade of the neurotoxicity of the peptide assembly in a concentration-dependent manner (Figure 2(a)), which is in good agreement with our previous finding [17]. Regarding this action, *in vitro* experiments demonstrated that conformation-dependent antibodies [30–35] and their fragments [28] successfully immunoneutralized the toxicity of A*β*Os. Presently, there is no evidence that antibody-A*β*O interactions induce nontoxic conformational changes. In our previous experiment [17], sortilin is upregulated in the presence of A*β*Os, and sortilin-p75^NTR^ receptors are formed on neuronal membranes; however, the downregulation of sortilin and the dissociation of sortilin from p75^NTR^ occur by the direct sequestration of A*β*Os in the presence of 72D9. Through this mechanism, extracellular A*β*Os appear to be maintained in a nontoxic state when complexed with 72D9. 

To further assess the above issue, we reevaluated the brains of the mice with improved cognition that received 72D9 immunotherapy [17]. Of note, we found that 72D9 decorated neurons in the brain parenchyma of 3x-Tg AD mice at 26 months of age (Figure 2(b)); this was not the case in the control IgG2b-immunized 3x-Tg AD mice of the same age (Figure 2(c)). Thus, some 72D9 got across BBB and directly immunoneutralized A*β*Os in the brain parenchyma. Triple labeling analysis revealed that 72D9 and A11 immunofluorescences overlap in the cytosol of neurons, indicating that 72D9 can be internalized into neurons together with A*β*Os (Figure 2(b)). Tampellini et al. [36] showed that anti-A*β* antibodies bind to the extracellular A*β* domain of the amyloid precursor protein (APP) and are internalized together with APP, followed by the clearance of intraneuronal A*β* via the endosomal-lysosomal pathway. Since 72D9 does not cross-react with APP [17], another yet unknown mechanism drives this internalization. Of note, most of the 72D9-negative pyramidal neurons exhibited atypical, eccentric large nuclei with abnormal chromatin morphology and distributions, features indicative of impending neuronal degeneration (Figure 2(e)). Such abnormalities were less evident in the 72D9-positive pyramidal neurons (Figure 2(d)), indicating that internalized A*β*O as a complex with 72D9 appears to be maintained in a nontoxic state. Although the precise mechanisms for resolving this issue should be clarified in future studies, note that 72D9 can sequester both extracellular and intraneuronal A*β*Os in a nontoxic state. 

## 4. Conclusions

Because A*β*O immunotherapy is promising for preemptive disease modifying therapy, research aimed at elucidating the molecular mechanisms underlying the action of A*β*Os and/or antibodies targeting A*β*Os is clearly required. The purpose of our study was to evaluate this issue. We herein found that an anti-A*β*O antibody plays an important role in the A*β* aggregation pathway in a chaperone-like manner and the intracerebral sequestration of A*β*Os in a nontoxic state, which is responsible for neuronal protection.
